# Department of Surgery

**Published:** 1901-04

**Authors:** W. E. A. Wyman, J. A. Sloan, Robert Jay

**Affiliations:** Milwaukee, Wis.; San Francisco, Cal.; West Liberty, Ia.


### DEPARTMENT OF SURGERY.

By W. E. A. Wyman, M.D.V., V.S.,
MILWAUKEE, WIS.

J. A. Sloan, B.Sc., M.D.V.,
SAN FRANCISCO, CAL.

Robert Jay, M.D.V.,
WEST LIBERTY, IA.

### Hoof Diseases.

(Continued from page 166.)

### Pododermatitis.

In those cases of inflammation with accumulation of exudates and sluggish resorption and subsequent imperfect filling out of the cavity with new horn resting upon the fleshy laminæ, there follows in time the formation of a keraphyllocele, since the newly-formed scar tissue resting upon the villi prevents the primary and secondary laminæ, previously distorted by the exudates, from taking their original course. Induration of the pododerm starting at the stratum vasculosum, decreasing toward the laminæ, which appear shortened and thickened, accompanies chronic aseptic pododermatitis.

Pressure atrophy of the os pedis is met with in the larger keraphylloceles. A peculiar ringed appearance follows pododermatitis, while the tree-bark-like covering of the horny box suggests pathological changes due to pododermatitis of the coronary cushion; the dry, cheesy nature of the horn along the white line, horny sole or frog, is the result of pathological changes in their respective originators, viz., fleshy sole, frog, or sensitive laminæ.

Supporting leg lameness is a prominent symptom of serous-pododermatitis. The animal points forward and moves the lame leg to and fro from time to time. Naturally, lameness increases on hard ground and going down hill, as is the case in almost all hoof-lameness, the intracapsular pressure being increased, and thus pain. The pain in the pododerm is marked; remember that the swollen parts are wedged in between two solid, practically unyielding layers. Palpation and percussion with the pincers and hammer will give interesting data. Increased heat of the horny box is readily detected by palpating the lame member with the hand, especially if the temperature of the diseased member is compared with that of the sound one, bearing in mind those points which lead to errors fully dealt with in my work on the *Clinical Diagnosis of Lameness.* Inspection of the diseased parts reveals swelling of the coronary region, especially of the bulbs of the heels, the only parts capable of visible swelling, the pododerm otherwise being confined within the unyielding capsule—the hoof. Palpation of the swollen heel exhibits some fluctuation, representing the accumulated transudate which when sufficient in amount may lead to separation of the perioplic band. Depending on the degree of pododermatitis present the heels may become dislocated, bulge out, and, while eventually returning to a normal state, the horn formation, usually excessive during that time, remains, and the foundation to coronary contraction is laid, so commonly seen in weak-heeled, long-toed hoofs.

Increased pulsation of the metacarpal arteries, so often mentioned to represent the decisive symptom of acute inflammatory changes within the hoof, is present, together with more or less œdema, extending at times half the way up the shin—the results of disturbed circulation.

To positively diagnose serous pododermatitis the sole should be pared out, when, especially at the inflection of the bars, the characteristic of serous pododermatitis appears, namely, the peculiar glassy, translucent, fatty, glistening and yellow color of the horn of that region depending upon the imbibition of the serous or serofibrinous exudate, while naturally less prominent in the strongly pigmented hoof.

In the chronic form the lameness is less marked, but such hoofs are subject to acute attacks. In the chronic form no œdema and a slightly increased pulsation demand a close examination to give a correct diagnosis, or the common error of navicular arthritis is indulged in. The yellow color of the sole horn is always present in the acute form, while the eccentric rings about the hoof, the dry, cheesy horn along the white line or in the horny sole or frog are met with in the chronic form, the solid union of the parts having been interfered with by the inflammatory process. A loose wall is a common sequel to serous pododermatitis.

The acute form may be confounded with hemorrhagic and suppurative pododermatitis. In the hemorrhagic form on paring the sole no glassy, translucent, yellow coloration is observed, but instead of it a diffused, reddish color, while in the suppurative pododermatitis black spots with pus underneath and a strongly beating metacarpal pulse are characteristic.

(To be continued.)

